# The Royal College of Ophthalmologists National Ophthalmology Database age-related macular degeneration (AMD) audit: report 1, associations with socio-economic deprivation in neovascular AMD

**DOI:** 10.1038/s41433-026-04382-8

**Published:** 2026-03-24

**Authors:** Riddhi Shenoy, Mable T. Monachan, Marta Gruszka-Goh, Martin McKibbin

**Affiliations:** 1 Ulverscroft Eye Unit, University of Leicester, Leicester, United Kingdom; 2 Gloucestershire Hospitals NHS Foundation Trust, Cheltenham, UK; 3 The Royal College of Ophthalmologists’ National Ophthalmology Database Audit, London, UK; 4 Leeds Teaching Hospitals NHS Trust, Leeds, UK

**Keywords:** Epidemiology, Scientific community

## Abstract

**Background:**

Early diagnosis and treatment of neovascular age-related macular degeneration (NvAMD) improve vision outcomes. This analysis investigates associations of English indices of multiple deprivation 2019 (IMD2019) with baseline characteristics, key care processes and visual acuity (VA) outcomes for NvAMD in the National Ophthalmology Database (NOD).

**Methods:**

Eligible eyes started treatment for NvAMD in England between 01/04/2020 and 31/03/2023. Participating centres with ≥25 eyes with baseline VA and IMD2019 data were included.

**Results:**

Eligible for analysis were 48,583 eyes from 60 English centres. Between decile 1 (most deprived) and decile 10 (least deprived), median age at start of treatment ranged from 79 to 82 years and median baseline VA ranged from 56 to 60 ETDRS letters. After one year of treatment (−28 to +84 days), the median number of injections administered ranged from 7 to 8 across deciles. Loss to follow-up was observed in 13.7% eyes in decile 1, and 11.8% in decile 10. Median VA at 12 months ranged from 61 to 65 ETDRS letters across deciles. A “good” VA outcome (≥70 ETDRS letters) was achieved by 45.5% in decile 10, compared with 35.9% observed in decile 1 (*p* < 0.001). A “poor” VA outcome (≥10 ETDRS letter loss from baseline) occurred in 18.4% of eyes in decile 1 versus 14.5% in decile 10 (*p* < 0.001).

**Conclusions:**

Patients starting NHS-funded treatment in England for NvAMD and living in areas of higher socioeconomic deprivation were typically younger, had lower baseline acuity and achieved worse VA outcomes than those from lower deprivation areas, with little variation in treatment between the deciles.

## Introduction

Age-related macular degeneration (AMD) is a chronic progressive eye condition and the leading cause of irreversible visual impairment in older people in developed countries, with 200 million people estimated to have AMD worldwide in 2020 [1, 2].

Neovascular AMD (NvAMD) is an advanced form of AMD characterised by macular neovascularisation that can cause rapid central vision loss. Early diagnosis and treatment of NvAMD with intravitreal inhibition of vascular endothelial growth factor (VEGF) improves vision outcomes [3–5]. Notably, along with age, visual acuity (VA) at presentation has been shown to be one of the strongest predictors of long-term vision outcomes [6, 7].

Inequality in eye health is increasingly being identified across the conditions most responsible for visual impairment, with socioeconomic deprivation in the UK associated with more advanced eye disease and lower uptake of optometry services [8–11]. Existing evidence for the association between deprivation and AMD is inconsistent, though real-world data largely indicates disparities by socioeconomic status, with lower baseline vision and greater prevalence of AMD observed amongst those living in areas of greatest deprivation, and subsequent poorer visual acuity outcomes following treatment [12–15].

This study reports analysis of data supplied to The Royal College of Ophthalmologists (RCOphth) National Ophthalmology Database (NOD) AMD Audit, investigating associations of deprivation with baseline characteristics, key care processes and VA outcomes in NvAMD.

## Methods

The RCOphth NOD National AMD Audit is open to centres performing NHS-funded treatment of NvAMD within the UK and Channel Islands. The data is collected as part of routine clinical care on electronical medical record systems (EMR) or in-house databases and submitted annually for eyes starting treatment for NvAMD. Further information on the RCOphth NOD AMD Audit can be found on the audit website (www.nodaudit.org.uk).

Eligible for analysis were NHS-funded intravitreal anti-VEGF treatments submitted to RCOphth NOD administered in English centres with at least 25 treated eyes between 01/04/2020 and 31/03/2023 (Supplementary File 1 for list of participating centres). Inclusion criteria for eyes required recording of baseline VA and the English Indices of Multiple Deprivation (IMD) 2019 for each patient [16]. (IMD is a percentile rank estimate of relative deprivation that ranks neighbourhoods in England across seven domains (income, employment, education, health, crime, housing and living environment). Due to different measures of deprivation used in Scotland, Wales and Ireland, only data from England were analysed.) During data extraction, IMD deciles were matched to the patient’s post code, from decile 1 (most deprived) to decile 10 (least deprived), and transferred to the RCOphth NOD without the patient’s postcode. This analysis reports outcomes over the first 12 months of eyes commencing treatment in the 2020–2022 NHS years.

### Outcome measures

VA at baseline and after 12 months of treatment (−28 to +84 days) was recorded in ETDRS letters. A change of five ETDRS letters is equivalent to 0.10 LogMAR unit. A good” VA outcome at 12 months was defined as ≥70 ETDRS letters. A “poor” VA outcome at 12 months was defined as a decrease of ≥10 ETDRS letters from baseline. Eyes with a baseline VA ≤25 ETDRS letters were excluded from poor VA outcome analysis (Supplementary Fig. 1). Chi-squared test was used to compare “good” and “poor” visual outcomes between decile1 and decile10.

Key care processes included starting treatment within 14 days of referral from primary care, completing the first 3 injections during the loading phase of initial monthly injection within 10 weeks and the total number of injections administered to an eye during the first year of treatment.

Loss to follow-up was defined as eyes for which no clinical information was available at 12 months (+84 days) following treatment commencement. All eyes with VA data or injection data at one year were considered to have completed follow-up.

### Statistical analysis

All analyses were performed using STATA 18 (StataCorp. 2023. Stata Statistical Software: Release 18. College Station, TX: StataCorp LLC).

## Results

### Baseline characteristics

Eligible for analysis were 48,583 eyes of 42,043 patients from 60 English participating centres (including 47 NHS Trusts and 13 independent sector treatment centres). Anti-VEGF injections were administered to 24,001 (49.4%) left eyes and 24,582 (50.6%) right eyes. Of 42,043 patients, 25,240 (60.0%) were female, 15,514 (36.9%) were male, and the gender was not stated for 1289 (3.1%) patients.

The majority of the sample consisted of 33,161 (68.3%) first-treated eyes, of which the median age at start of treatment was 81 years (IQR: 75–86 years). The number of second-treated eyes was 9864 (20.3%), with a median age at start of treatment of 83 years (IQR: 77–87 years). Immediate sequential bilateral intravitreal treatment (ISBIVT) was given to 5558 (11.4%) eyes from 2779 patients, with a median age at start of treatment of 82 years (IQR: 76–87 years). The overall prevalence of diabetes mellitus was 12.7% (5327 patients).

### Baseline age and visual acuity by IMD decile

**Fig. 1:**
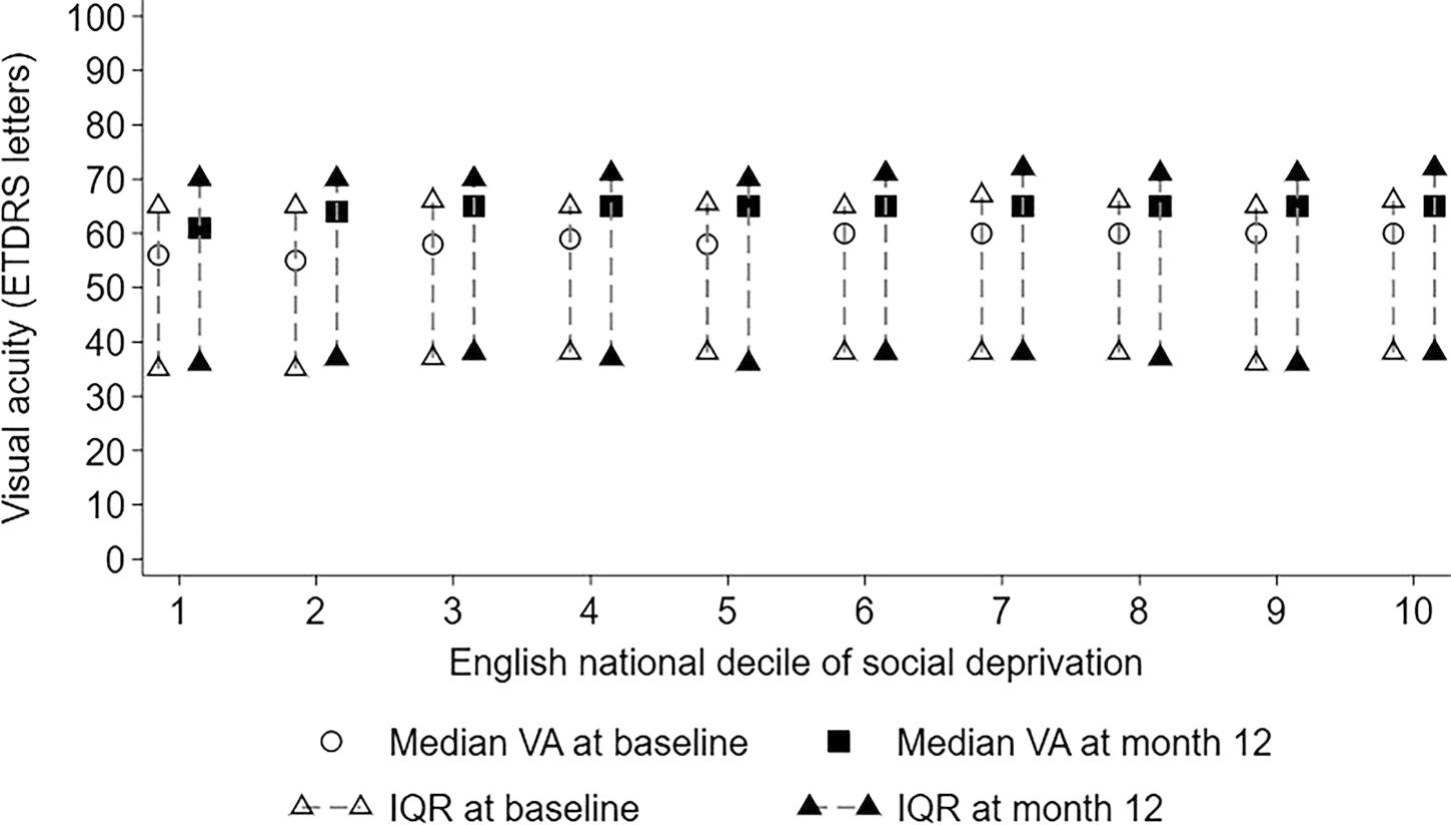
Median + IQR plots of ETDRS visual acuity (VA) at baseline (*N* = 48,583 treated eyes from 60 participating centres) and at one-year (*N* = 39,439 treated eyes from 58 participating centres) by national decile of social deprivation for England. The ‘line’ displays the inter-quartile range (IQR) with the median in the middle. The national decile of social deprivation indicates the most deprived decile (decile 1) up to the least deprived decile (decile 10) of England.

Median age was 79 years in decile 1 increasing to 82 years in decile 10 (Table 1). The median baseline VA overall was 60 ETDRS letters (IQR: 45–70 ETDRS letters), trending to decreasing baseline VA with increasing deprivation, with a median of 56 ETDRS letters in decile 1 and 60 ETDRS letters in decile 10 (Fig. 1). This trend was also present when analysing first-treated eyes only, but not in second-treated eyes. For first-treated eyes, the median baseline VA was 55 ETDRS letters (IQR: 40–68 ETDRS letters), where median baseline VA was 55 ETDRS letters in decile 1 increasing to 59 ETDRS letters in decile 10. For second-treated eyes, the median baseline VA was 65 ETDRS letters (IQR: 54–71 ETDRS letters), across all deciles.

**Table 1:** Patients’ characteristics, categorised baseline visual acuity (VA), and intravitreal injections for decile of social deprivation.

variables	Decile of social deprivation									
	1	2	3	4	5	6	7	8	9	10
*N*	3222	3432	3690	4291	4830	5098	5506	5836	5956	6722
Age at 1st injection										
(years)										
Median	79	80	80	81	81	81	82	82	82	82
IQR	(73–85)	(74–86)	(74–85)	(75–86)	(75–87)	(76–86)	(76–87)	(76–87)	(77–87)	(77–87)
VA at baseline										
(Column %)										
≤25 letters	10	10.9	9	8.5	7.9	8.3	8.6	7.7	7.8	6.9
26–35 letters	9.8	10.7	9.5	8.9	9.2	8.9	8.4	7.9	8.2	7.5
36–55 letters	30.1	28.7	29.2	30.4	30.7	28.9	29.2	28	28.1	27.8
56–69 letters	25.9	25.6	26.5	26.1	26.5	28.4	26.8	28.6	27.9	28.8
≥70 letters	24.2	24.1	25.8	26.1	25.7	25.7	26.9	27.9	28	29.1
Completing the loading phase (column %)										
<3 injections	6	6.7	6.3	5.7	5.9	5.1	5.3	5.4	5.9	4.8
Within 10 weeks	71.3	69.8	69.5	70.9	70.6	72.7	71.6	72.4	73.5	72.4
10–12 weeks	9.2	9.9	9.8	9.7	10.1	10.4	9.9	9.1	8.7	9
12–16 weeks	7.1	7.1	7.1	6.3	6.2	5.6	6.2	6.4	5.7	6.2
4–6 months	4.5	4.2	4.5	4.9	4.4	3.9	4.3	4.3	4	4.8
>6 months	1.9	2.3	2.8	2.5	2.8	2.3	2.7	2.4	2.2	2.8
Injections over one year (*N* = 345,062)	22,535	23,447	25,203	29,446	33,725	36,318	39,473	42,271	42,838	49,806
Median (IQR) injection per eye	7 (5–9)	7 (5–9)	7 (5–9)	7 (5–9)	7 (5–9)	7 (5–9)	7 (5–9)	8 (5–9)	7 (5–9)	8 (5–9)
Lost to follow up after one year (Column %)	13.7	15.5	13.1	14.2	13.2	12.6	13.8	12.1	12.6	11.8

*N* = 48,583 treated eyes from 60 participating centres.

Treated eyes from less deprived deciles were more likely to have baseline VA of ≥70 ETDRS letters (29.1%) and less likely to have baseline VA of ≤ 25 ETDRS letters (6.9%) (Table 1).

#### Distribution of treated eyes across IMD deciles

**Fig. 2:**
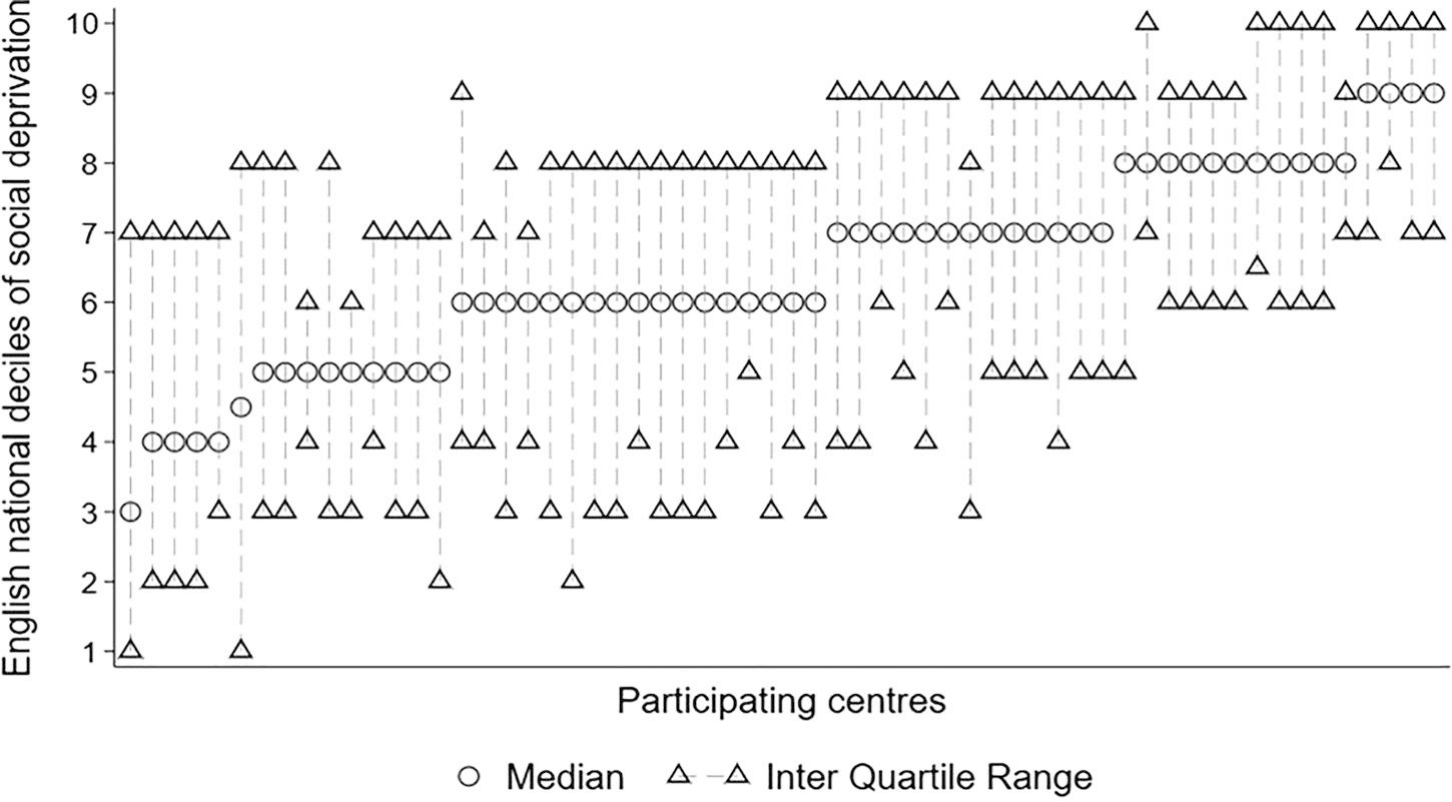
Median + IQR plots of decile of the social deprivation by participating centre and ordered by median decile. The ‘line’ displays the inter-quartile range (IQR) with the median in the middle. The national deciles of social deprivation indicate the most deprived (decile 1) up to the least deprived (decile 10) of England. *N* = 48,583 treated eyes from 60 participating centres.

The proportion of treated eyes from each IMD decile ranged from 6.6% to 13.8% for deciles 1–10. All bar 8 centres treated patients from every IMD decile. The median IMD decile from each centre ranged from decile 3 to decile 9 (Fig. 2).

From the sample, 43,754 (90.1%) of eyes treated in traditional NHS centres, representation from IMD deciles ranged from 6.3% (decile 1) to 13.9% (decile 10). Similarly, in the 4829 (9.9%) of eyes treated in the independent sector, representation ranged from 10.0% (decile 1) to 13.2% (decile 10).

### Intravitreal injection pathway uptake by IMD decile

Over the first year of treatment, 48,583 eyes received a total of 345,062 intravitreal injections, of which the proportion ranged from 6.4% to 13.8% for decile 1–10 (Table 1). Of the total cohort, only 21,836 eyes (44.9%) had a recorded referral date, and only 16.6% had a documented reason for referral. Due to these limitations in data completeness, results regarding initiation of treatment within 14 days of primary care referral are not presented, as poor data quality restricts their applicability to the entire cohort. The initial loading phase of three anti-VEGF injections was completed within 10 weeks of the first injection by 34,827 (71.7%) eyes (Table 1). The median number of anti-VEGF injections per eye was 7 (IQR: 5–9). There was little variation across IMD deciles for loading phase completion, and the median number of injections during the first year of treatment.

Overall, 6354 (13.1%) eyes were lost to follow up within one year of treatment and the proportion ranged between 13.7% to 11.8% from deciles 1 to 10, being highest in decile 2 (15.5%) (Table 1). While 456 (7.2%) eyes stopped treatment for clinical reasons, there were no reasons reported for 5008 (78.8%) eyes lost to follow-up.

### Visual acuity at 12 months of treatment by IMD decile

After one year, VA was recorded for 39,439 (81.2%) eyes from 58 centres. Median VA was 65 ETDRS letters (IQR: 48–75 ETDRS letters), trending towards increasing VA with decreasing deprivation, with median VA at 12 months 61 ETDRS letters (decile 1) and 65 ETDRS letters (decile 10) (Fig. 1). Differentiating between first and second-treated eyes showed similar trends. The median VA of first-treated eyes at 12 months was 60 ETDRS letters in decile 1 and 65 ETDRS letters in decile 10. Median VA at 12 months of second-treated eyes was 66 ETDRS letters in decile 1 and 70 ETDRS letters in decile 10 (Supplementary Tables 2, 3). The proportion of eyes in which VA improved by >5 ETDRS letters was 39.1%, by >10 ETDRS letters was 25% and by >15 ETDRS letters was 15%, with similar gains across deciles.

“Good” VA (≥70 ETDRS letters) at 12 months was achieved by 16,414 (41.6%) of all eyes, ranging from 35.9% (decile 1) to 45.5% (decile 10) (Table 2), with a significantly higher proportion of patients with “good” VA in the least deprived decile 10 compared to the most deprived decile 1 (*p* < 0.001).

**Table 2:** Categorised visual acuity (VA) at 12-month, Good VA (*N* = 39,439), and poor VA (*N* = 36,792) for decile of the social deprivation from 58 participating centres.

Variables	Decile of social deprivation									
	1	2	3	4	5	6	7	8	9	10
Number of eyes at one year (*N*)	2566	2676	2945	3401	3933	4186	4458	4811	4898	5565
VA at one year (column %)										
≤25 letters	9.5	10.2	9.2	8.2	8.9	8.6	8.3	7.8	7.9	7
26–35 letters	8	7.2	7.1	7.8	7.1	6.3	6.9	6.8	6.8	6.1
36–55 letters	22.2	20.8	20.4	20.3	21.2	20	18.6	19.4	18.5	19.5
56–69 letters	24.4	23.9	24.2	24	22.6	23.8	22.8	23.5	22.7	21.9
≥70 letters (Good vision)	35.9	37.9	39.1	39.7	40.2	41.3	43.4	42.5	44.1	45.5
Number of eyes at one year with baseline VA > 25 ETDRS letters	2 348	2 458	2 730	3 177	3 675	3 915	4 134	4 511	4 589	5 255
Poor vision (column %)	18.3	17.2	16	16.3	16.8	15.9	15.4	15.5	16	14.5

“Poor” VA outcome at 12 months (≥10 ETDRS letters decrease from baseline VA) was observed in 5879 (16.0%) of all eyes. This proportion was significantly higher among patients in the most deprived decile 1 (18.3%) compared to those in the least deprived decile 10 (14.5%) (*p* < 0.001) (Table 2).

## Discussion

This review of UK AMD Audit data collected as part of routine care over a 3-year period shows significant associations between socioeconomic deprivation and visual acuity (VA), both at baseline and at 12 months, for NHS-funded treatment of NvAMD in England. This relationship persists despite minimal differences in the audited aspects of the care pathway, suggesting that factors beyond clinical delivery may influence outcomes. The findings underscore a clear socioeconomic gradient: patients from the least deprived areas were significantly more likely to achieve “good” VA at 12 months, while those from the most deprived areas were significantly more likely to experience “poor” VA at 12 months. Additionally, a trend toward greater loss to follow-up among patients in more deprived areas further highlights potential barriers to sustained engagement with care.

Median baseline VA was lower in patients from the areas of greatest deprivation and 12-month VA outcomes were also worse, despite these patients being younger at the start of treatment. This is in contrast with published studies that generally report better visual outcomes amongst younger patients and may suggest deprivation is associated with more advanced AMD at presentation [17, 18]. This may further indicate barriers to access to diagnosis of AMD in areas of deprivation that are reflected in findings from previous UK studies [12, 19]. One single centre retrospective study in the UK (*n* = 756) reported that people living in the most deprived areas were significantly more likely, when compared to those from the least deprived areas, to present with severe reduction in acuity, after adjusting for age, gender and distance from treatment centre (OR  =  4.07; 95% Cl = 1.50–11.0; *P*  =  0.006,) [12]. Similar associations were reported in another single centre retrospective study in Birmingham (*n* = 120), but were not found in a similar study in Glasgow (*n* = 240). However, significant limitations of these retrospective studies include variable measures of deprivation used, variable inclusion of relevant confounders and small sample sizes [12, 19, 20].

Comparing first and second-treated eyes, the trend to worse baseline VA with greater deprivation was only seen in first-treated eyes, suggesting that patients developing NvAMD in their second eye, likely identified through monitoring in secondary care, may not experience the same initial barriers to access. However, worse 12-month VA was seen in both first and second-treated eyes, suggesting that socio-economic deprivation may have an independent association with acuity outcomes, rather than simply being a surrogate marker of baseline VA. Across all treated eyes, 12-month VA outcomes in this audit remained worse in the eyes of patients from areas of highest deprivation.

Barriers to access to primary care, where patients with AMD may first present, may contribute to the association seen with deprivation and worse baseline VA. Analysis of NHS digital data has shown substantial variation in the provision of optometry services in England with much lower rates in more deprived areas versus more affluent areas [11]. Inequalities in uptake of eye examinations have also been shown, with analysis of General Ophthalmic Services (GOS) claims for eye examinations in the UK showing people aged over 60 years living in the least deprived areas being 15–71% more likely to access an NHS-funded eye examination compared to those in the same age group living in the most deprived areas [8, 21]. Similar trends are reported globally, with studies in the USA and Canada reporting lower uptake of eye services amongst people with lower education and income levels, current smokers, Black ethnic groups and those older than 65 years, with reasons for not seeking eye care including no insurance or cost of examination and lack of perceived eye problem [22, 23].

Observational studies of real-world data, including the RAINBOW and PERSEUS studies, have consistently shown the association of regular treatment and prompt completion of the loading phase of treatment with better greater acuity outcomes and the same findings have been replicated in the annual reports of the UK AMD audit [24, 25]. In this audit, 71.7% of eyes overall completed the first 3 injections during the loading phase of treatment within 10 weeks, with little variation across IMD deciles. This is comparable to another observational study in the UK reporting over 75% completion within 10 weeks (*n* = 7 686), which also reported worse VA with an incomplete loading phase of treatment by LogMAR 0.078 (95% CI 0.039–0.12), approximately four ETDRS letters, when compared with eyes with fast loading phase completion [14]. There was also little variation in the median number of intravitreal injections by IMD decile. The audited key care processes, therefore, did not show notable differences by socioeconomic deprivation, in contrast to other national audits [26]. Further research is warranted to identify specific components of the treatment pathway that may vary by socioeconomic deprivation. Such insights could inform future audits of AMD service outcomes stratified by deprivation and guide the design, implementation, and evaluation of interventions aimed at reducing health inequalities.

Trends towards greater loss to follow-up were observed in the most deprived deciles in this analysis, which has not been reported in other UK studies of AMD. This may be associated with other variables not included in this analysis [12, 19]. Studies in the USA investigating loss to follow up in AMD services have identified other associations including older age, Black or Asian ethnicity, non-English speaking groups and comorbidities such as diabetes or dementia [27–30]. Factors increasing rates of loss to follow-up in treatment of wet AMD have significant implications on increasing vision loss, and poor baseline VA itself has been linked to increased loss to follow up, suggesting persistent barriers to access throughout the care pathway [7, 31, 32]. Distance from home to hospital is also suggested as a reason for loss to follow up in treatment of AMD and other conditions affecting older people, perhaps linked to identifying an escort to the appointment [33, 34]. Other factors influencing adherence in qualitative studies include logistic barriers, such as booking appointments, and psychological barriers surrounding experience of receiving intravitreal injections [32, 35, 36].

Strengths of this study include the contribution of data over three years from an estimated 50% of all eligible eyes in England, comprising one of the largest real-world databases of intravitreal injections for NvAMD, thereby increasing the generalisability of findings. Limitations include the use of only English centres as deprivation is measured differently in Scotland, Wales and Northern Ireland. Furthermore, deprivation data were available for centres using Medisoft or mediSIGHT electronic medical records, but not available from centres using OpenEyes software. Data quality relating to the duration of symptoms and recording of the date of initial referral was poor so that no analysis of delayed presentation to primary care colleagues or delays in starting treatment after diagnosis were possible. Analysis also included the use of two eyes per person introducing patient-level correlation.

This audit has found significant associations between higher socioeconomic deprivation and worse 12-month VA, despite the patients being younger at the start of treatment and no clear difference in the elements of the care pathway studied. Moreover, this difference in outcome was present in both first and second-treated eyes, despite there being no difference in the baseline VA of second-treated eyes by decile of deprivation. Further exploration could seek to explain the association between greater deprivation and poorer vision outcomes across both first-treated eyes, where differences in baseline VA exist across IMD deciles, and second-treated eyes, where there is no difference in baseline VA by decile. This could underpin further investigation of barriers to access to AMD services amongst underserved populations.

## Summary

### What was known before

- Reducing inequity in eye health is a national priority and links between socioeconomic deprivation and advanced eye disease are already described.
- Few studies in the UK have investigated associations between socio-economic deprivation and baseline characteristics, age, key care processes and visual acuity outcomes after treatment for neovascular age-related macular degeneration.

### What this study adds

- Socio-economic deprivation is associated with age and visual acuity at presentation, key care processes and visual acuity outcomes after NHS-funded treatment in England for neovascular age-related macular degeneration.
- Further analysis is required to investigate if socioeconomic deprivation is independently associated with baseline visual acuity and outcomes after treatment.

## Supplementary information

Supplementary files description

Supplementary file 1

Supplementary Figure 1

Supplementary Tables 2 and 3

## Data Availability

The data that support the findings of this study are available from The Royal College of Ophthalmologists, but restrictions apply to the availability of these data, which were used under license for the current study, and so are not publicly available. Data are howeve,r available from the authors upon reasonable request and with permission of The Royal College of Ophthalmologists

## References

[1] Furtado JM, Jonas JB, Tapply I, Fernandes AG, Cicinelli MV, Arrigo A, et al. Global estimates on the number of people blind or visually impaired by age-related macular degeneration: a metaanalysis from 2000 to 2020. Eye. 2024;38:2070–82. 38965321 10.1038/s41433-024-03050-z PMC11269688

[2] Wong WL, Su X, Li X, Cheung CMG, Klein R, Cheng C-Y, et al. Global prevalence of agerelated macular degeneration and disease burden projection for 2020 and 2040: a systematic review and meta-analysis. Lancet Glob Health. 2014;2:e106–e16. 25104651 10.1016/S2214-109X(13)70145-1

[3] Lim JH, Wickremasinghe SS, Xie J, Chauhan DS, Baird PN, Robman LD, et al. Delay to treatment and visual outcomes in patients treated with anti-vascular endothelial growth factor for age-related macular degeneration. Am J Ophthalmol. 2012;153:678–86.e2. 22245460 10.1016/j.ajo.2011.09.013 PMC4869322

[4] Weber M, Dominguez M, Coscas F, Faure C, Baillif S, Kodjikian L, et al. Impact of intravitreal aflibercept dosing regimens in treatment-naïve patients with neovascular age-related macular degeneration: 2-year results of RAINBOW. BMC Ophthalmol. 2020;20:206. 32450838 10.1186/s12886-020-01468-z PMC7249443

[5] Schmidt-Erfurth U, Chong V, Loewenstein A, Larsen M, Souied E, Schlingemann R, et al. Guidelines for the management of neovascular age-related macular degeneration by the European Society of Retina Specialists (EURETINA). Br J Ophthalmol. 2014;98:1144–67. 25136079 10.1136/bjophthalmol-2014-305702 PMC4145443

[6] Ho AC, Albini TA, Brown DM, Boyer DS, Regillo CD, Heier JS. The potential importance of detection of neovascular age-related macular degeneration when visual acuity is relatively good. JAMA Ophthalmol. 2017;135:268–73. 28114653 10.1001/jamaophthalmol.2016.5314

[7] Mollan SP, Fu DJ, Chuo C-Y, Gannon JG, Lee WH, Hopkins JJ, et al. Predicting the immediate impact of national lockdown on neovascular age-related macular degeneration and associated visual morbidity: an INSIGHT Health Data Research Hub for Eye Health report. Br J Ophthalmol. 2023;107:267–74. 34518162 10.1136/bjophthalmol-2021-319383 PMC9887382

[8] Shickle D, Farragher TM, Davey CJ, Slade SV, Syrett J. Geographical inequalities in uptake of NHS funded eye examinations: Poisson modelling of small-area data for Essex, UK. J Public Health. 2017;40:e171–e9. 10.1093/pubmed/fdx058 PMC6051445 28633479

[9] England L, O’Connor A. Do socioeconomic inequalities exist within ophthalmology and orthoptics in the UK? A scoping review. Br Ir Orthopt J. 2024;20:31–47. 38250169 10.22599/bioj.338 PMC10798172

[10] Wong TL, Ang JL, Deol S, Buckmaster F, McTrusty AD, Tatham AJ. The relationship between multiple deprivation and severity of glaucoma at diagnosis. Eye. 2023;37:3376–81. 36959313 10.1038/s41433-023-02508-w PMC10035976

[11] Harper RA, Hooper J, Fenerty CH, Roach J, Bowen M. Deprivation and the location of primary care optometry services in England. Eye. 2024;38:656–8. 37770531 10.1038/s41433-023-02774-8 PMC10920810

[12] More P, Almuhtaseb H, Smith D, Fraser S, Lotery AJ. Socio-economic status and outcomes for patients with age-related macular degeneration. Eye. 2019;33:1224–31. 30858565 10.1038/s41433-019-0393-3 PMC7005865

[13] Yip JL, Khawaja AP, Chan MP, Broadway DC, Peto T, Luben R, et al. Area deprivation and age related macular degeneration in the EPIC-Norfolk eye study. public health. 2015;129:103–9. 25687711 10.1016/j.puhe.2014.10.012 PMC4357435

[14] Relton SD, Chi GC, Andrew L, West RM, Martin M, Santiago C, et al. Associations with visual acuity outcomes after 12 months of treatment in 9401 eyes with neovascular AMD. BMJ Open Ophthalmol. 2022;7:e001038. 36161843 10.1136/bmjophth-2022-001038 PMC9240889

[15] Relton SD, Chi GC, Lotery AJ, West RM. Real world AMDtoEMRUG, McKibbin M. Associations with baseline visual acuity in 12,414 eyes starting treatment for neovascular AMD. Eye. 2023;37:1652–8. 36028762 10.1038/s41433-022-02208-x PMC10219991

[16] Ministry of Housing CLG. English indices of deprivation 2019 (IoD2019). Ministry of Housing CLG; 2019.

[17] Rosenfeld PJ, Shapiro H, Tuomi L, Webster M, Elledge J, Blodi B. Characteristics of patients losing vision after 2 years of monthly dosing in the phase III ranibizumab clinical trials. Ophthalmology. 2011;118:523–30. 20920825 10.1016/j.ophtha.2010.07.011

[18] Ying G-s, Huang J, Maguire MG, Jaffe GJ, Grunwald JE, Toth C, et al. Baseline predictors for one-year visual outcomes with ranibizumab or bevacizumab for neovascular age-related macular degeneration. Ophthalmology. 2013;120:122–9. 23047002 10.1016/j.ophtha.2012.07.042 PMC3536921

[19] Sharma HE, Mathewson PA, Lane M, Shah P, Glover N, Palmer H, et al. The role of social deprivation in severe neovascular age-related macular degeneration. Br J Ophthalmol. 2014;98:1625–8. 24997180 10.1136/bjophthalmol-2014-304959

[20] Acharya N, Lois N, Townend J, Zaher S, Gallagher M, Gavin M. Socio-economic deprivation and visual acuity at presentation in exudative age-related macular degeneration. Br J Ophthalmol. 2009;93:627–9. 19091850 10.1136/bjo.2008.147231

[21] Shickle D, Farragher TM. Geographical inequalities in uptake of NHS-funded eye examinations: small area analysis of Leeds, UK. J Public Health. 2015;37:337–45. 10.1093/pubmed/fdu039 25015580

[22] Sherman E, Niziol LM, Hicks PM, Johnson-Griggs M, Elam AR, Woodward MA, et al. A screening strategy to mitigate vision impairment by engaging adults who underuse eye care services. JAMA Ophthalmol. 2024;142:909–16. 39172473 10.1001/jamaophthalmol.2024.3132 PMC11342220

[23] Aljied R, Aubin MJ, Buhrmann R, Sabeti S, Freeman EE. Eye care utilization and its determinants in Canada. Can J Ophthalmol. 2018;53:298–304. 29784169 10.1016/j.jcjo.2018.01.021

[24] Michel W, Laurent K, Florence C, Céline F, Isabelle A, Ingrid D, et al. Impact of intravitreal aflibercept dosing regimens in treatment-naÃ¯ve patients with neovascular age-related macular degeneration in routine clinical practice in France: results from the RAINBOW study. BMJ Open Ophthalmol. 2020;5:e000377. 10.1136/bmjophth-2019-000377 PMC7254134 32518833

[25] Framme C, Eter N, Hamacher T, Hasanbasic Z, Jochmann C, Johnson KT, et al. Aflibercept for patients with neovascular age-related macular degeneration in routine clinical practice in Germany: twelve-month outcomes of PERSEUS. Ophthalmol Retin. 2018;2:539–49. 10.1016/j.oret.2017.09.017 31047606

[26] National Prostate Cancer Audit (NPCA) State of the Nation Report. London: National Cancer Audit Collaborating Centre, Royal College of Surgeons of England, 2025.

[27] Hamedani AG, Chang AY, Chen Y, VanderBeek BL. Disparities in glaucoma and macular degeneration healthcare utilization among persons living with dementia in the United States. Graefes Arch Clin Exp Ophthalmol. 2024;262:3947–55. 38995351 10.1007/s00417-024-06573-z PMC11608381

[28] Khurana RN, Li C, Lum F. Loss to follow-up in patients with neovascular age-related macular degeneration treated with anti–VEGF therapy in the United States in the IRIS® registry. Ophthalmology. 2023;130:672–83. 36858288 10.1016/j.ophtha.2023.02.021

[29] Nguyen AH, Davoudi S, Dong K, Bains A, Ness S, Subramanian ML, et al. Socioeconomic disparities in patients receiving intravitreal injections for age-related macular degeneration amid the COVID-19 pandemic. J Vitreoretina Dis. 2023;7:376–81. 10.1177/24741264231173771 PMC10311364 37701269

[30] Obeid A, Gao X, Ali FS, Aderman CM, Shahlaee A, Adam MK, et al. Loss to follow-up among patients with neovascular age-related macular degeneration who received intravitreal antivascular endothelial growth factor injections. JAMA Ophthalmol. 2018;136:1251–9. 30352121 10.1001/jamaophthalmol.2018.3578 PMC6583857

[31] Kusenda P, Caprnda M, Gabrielova Z, Kukova N, Pavlovic S, Stefanickova J. Understanding loss to follow-up in AMD patients receiving VEGF inhibitor therapy: associated factors and underlying reasons. Diagnostics. 2024;14:400. 10.3390/diagnostics14040400 PMC10887977 38396439

[32] Polat O, İnan S, Özcan S, Doğan M, Küsbeci T, Yavaş GF, et al. Factors affecting compliance to intravitreal anti-vascular endothelial growth factor therapy in patients with agerelated macular degeneration. Turkish J Ophthalmol Turk Oftalmol Derg. 2017;47:205–10. 10.4274/tjo.28003 PMC5563548 28845324

[33] Boulanger-Scemama E, Querques G, About F, Puche N, Srour M, Mane V, et al. Ranibizumab for exudative age-related macular degeneration: a five year study of adherence to follow-up in a real-life setting. J Fr Ophtalmol. 2015;38:620–7. 25913443 10.1016/j.jfo.2014.11.015

[34] Meer EA, Targ S, Zhang N, Hoggatt KJ, Mehta KM, Brodie F. Age-related macular degeneration injection frequency: effects of distance traveled and travel support. Retina. 2024;44:230–6. 37756667 10.1097/IAE.0000000000003947

[35] Müller S, Junker S, Wilke T, Lommatzsch A, Schuster AK, Kaymak H, et al. Questionnaire for the assessment of adherence barriers of intravitreal therapy: the ABQ-IVT. Int J Retin Vitreous. 2021;7:43. 10.1186/s40942-021-00311-x PMC8170736 34078475

[36] Giocanti-Aurégan A, García-Layana A, Peto T, Gentile B, Chi GC, Mirt M, et al. Drivers of and barriers to adherence to neovascular age-related macular degeneration and diabetic macular edema 3 treatment management plans: a multi-national qualitative study. Patient Pref Adherence. 2022;16:587–604. 10.2147/PPA.S347713 PMC8901255 35264847

