# Supplementary material for: The Royal College of Ophthalmologists National Ophthalmology Database age-related macular degeneration (AMD) audit: report 1, associations with socio-economic deprivation in neovascular AMD
Source: Eye (Lond). 2026 Mar 24;40(7):999–1004. doi: 10.1038/s41433-026-04382-8 (PMC13161383; doi:10.1038/s41433-026-04382-8)
Supplement: Supplementary file 1 — Supplementary files description [file 41433_2026_4382_MOESM1_ESM.docx]

Supplementary file 1: List of participating centres (doc)

Supplementary Figure 1: Flowchart of included eyes with inclusion and exclusion criteria (doc)

Supplementary Table 2: 1-year outcome by decile, first treated eyes (doc)

Supplementary Table 3: 1-year outcome by decile, second treated eyes (doc)
