# Supplementary material for: The Royal College of Ophthalmologists National Ophthalmology Database age-related macular degeneration (AMD) audit: report 1, associations with socio-economic deprivation in neovascular AMD
Source: Eye (Lond). 2026 Mar 24;40(7):999–1004. doi: 10.1038/s41433-026-04382-8 (PMC13161383; doi:10.1038/s41433-026-04382-8)
Supplement: Supplementary file 2 — Supplementary file 1 [file 41433_2026_4382_MOESM2_ESM.docx]

Supplementary file 1: List of participating centres

NHS Trusts in England:

Barking, Havering and Redbridge University Hospitals NHS Trust; Barts Health NHS Trust; Bradford Teaching Hospitals NHS Foundation Trust; Buckinghamshire Healthcare NHS Trust; Calderdale and Huddersfield NHS Foundation Trust; Chesterfield Royal Hospital NHS Foundation Trust; County Durham and Darlington NHS Foundation Trust; East Cheshire NHS Trust; East Sussex Healthcare NHS Trust; Epsom and St Helier University Hospitals NHS Trust; Gloucestershire Hospitals NHS Foundation Trust; Great Western Hospitals NHS Foundation Trust; Harrogate and District NHS Foundation Trust; Hull University Teaching Hospitals NHS Trust; Isle of Wight NHS Trust; Kettering General Hospital NHS Foundation Trust; King's College Hospital NHS Foundation Trust; Leeds Teaching Hospitals NHS Trust; Liverpool University Hospitals NHS Foundation Trust; London North West University Healthcare NHS Trust; Mid Cheshire Hospitals NHS Foundation Trust; Mid Yorkshire Teaching NHS Trust; Mid and South Essex NHS Foundation Trust; Moorfields Eye Centre at Bedfordshire Hospitals NHS Foundation Trust; Moorfields Eye Hospital NHS Foundation Trust*; North West Anglia NHS Foundation Trust; Oxford University Hospitals NHS Foundation Trust; Royal Berkshire NHS Foundation Trust; Royal Cornwall Hospitals NHS Trust; Royal Devon University Healthcare NHS Foundation Trust; Royal Free London NHS Foundation Trust; Royal United Hospitals Bath NHS Foundation Trust; Salisbury NHS Foundation Trust; Sheffield Teaching Hospitals NHS Foundation Trust; Somerset NHS Foundation Trust; South Warwickshire University NHS Foundation Trust; Surrey and Sussex Healthcare NHS Trust; The Hillingdon Hospitals NHS Foundation Trust; The Newcastle upon Tyne Hospitals NHS Foundation Trust; The Princess Alexandra Hospital NHS Trust; University Hospital Southampton NHS Foundation Trust; University Hospitals Birmingham NHS Foundation Trust; University Hospitals Bristol and Weston NHS Foundation Trust; Warrington and Halton Teaching Hospitals NHS Foundation Trust; Wirral University Teaching Hospital NHS Foundation Trust; Wrightington, Wigan and Leigh NHS Foundation Trust; York and Scarborough Teaching Hospitals NHS Foundation Trust.

Independent Sector Treatment Sites:

2 Sites from Optegra Eye Health Care: Manchester; Yorkshire

3 sites from Practice Plus Group: Southampton; Rochdale; Gillingham

8 sites from SpaMedica: Birmingham; Chelmsford; Coventry; Manchester; Newark; Romford; Solihull; West Lancashire

*Only data from Croydon Health services NHS Trust as the ophthalmology service in this trust is under the governance of Moorfields Eye Hospitals NHS Foundation Trust
