# Supplementary material for: The Royal College of Ophthalmologists National Ophthalmology Database age-related macular degeneration (AMD) audit: report 1, associations with socio-economic deprivation in neovascular AMD
Source: Eye (Lond). 2026 Mar 24;40(7):999–1004. doi: 10.1038/s41433-026-04382-8 (PMC13161383; doi:10.1038/s41433-026-04382-8)
Supplement: Supplementary file 3 — Supplementary Figure 1 [file 41433_2026_4382_MOESM3_ESM.docx]

Supplementary Figure 1: Flowchart of included eyes in the analysis with inclusion and exclusion criteria. National Ophthalmology Database (NOD), Age-related Macular Degeneration (AMD), Indices of Multiple Deprivation (IMD), Visual Acuity (VA)

**72 661 eyes**

**84 centres**

Centres submitted data to NOD AMD Audit (2020 – 2022 NHS years)

Eyes with IMD decile data in England

**51 872 eyes**

**71 centres**

Eyes with recorded baseline VA measurement

**48 766 eyes**

**71 centres**

**48 583 eyes**

**60 centres**

Eyes with VA recorded after 12 months of treatment and centres with ≥25 eligible eyes

**36 792 eyes**

**58 centres**

**39 439 eyes**

**58 centres**

Poor VA

Good VA

Centres with ≥25 eligible eyes

Eyes with >25 ETDRS letters at baseline and centres with ≥25 eligible eyes
