# Supplementary material for: The Royal College of Ophthalmologists National Ophthalmology Database age-related macular degeneration (AMD) audit: report 1, associations with socio-economic deprivation in neovascular AMD
Source: Eye (Lond). 2026 Mar 24;40(7):999–1004. doi: 10.1038/s41433-026-04382-8 (PMC13161383; doi:10.1038/s41433-026-04382-8)
Supplement: Supplementary file 4 — Supplementary Tables 2 and 3 [file 41433_2026_4382_MOESM4_ESM.docx]

Supplementary Table 2: Visual acuity (VA) outcome at one year of First treated eyes over deciles

| Deciles | Number of eyes at one year | Median (IQR) VA  (ETDRS letters) | Good VA (Row %) | Number of eyes at one year (>25 letters) | Poor VA (Row %) |
| --- | --- | --- | --- | --- | --- |
| 1 | 537 | 66 (50 - 75) | 46.0 | 511 | 21.5 |
| 2 | 500 | 66 (52 - 75) | 45.8 | 480 | 17.5 |
| 3 | 585 | 69 (55 - 75) | 49.1 | 562 | 16.7 |
| 4 | 717 | 67 (55 - 75) | 46.6 | 702 | 16.7 |
| 5 | 828 | 66.5 (55 - 75) | 46.5 | 796 | 19.2 |
| 6 | 917 | 69 (55 - 75) | 49.1 | 880 | 15.9 |
| 7 | 986 | 70 (55 - 75) | 50.4 | 953 | 16.7 |
| 8 | 1 031 | 69 (55 - 75) | 48.8 | 1 003 | 17.5 |
| 9 | 1 039 | 69 (55 - 75) | 49.6 | 1 000 | 17.1 |
| 10 | 1 200 | 70 (55 - 75) | 53.3 | 1 161 | 15.0 |

| Deciles | Number of eyes at one year | Median (IQR) VA (ETDRS letters) | Good VA (Row %) | Number of eyes at one year (>25 letters) | Poor VA (Row %) |
| --- | --- | --- | --- | --- | --- |
| 1 | 1 775 | 60 (44 - 71) | 33.1 | 1 609 | 17.7 |
| 2 | 1 804 | 62 (45 – 73.5) | 36.4 | 1 629 | 16.8 |
| 3 | 1 994 | 63 (45 - 74) | 37.0 | 1 830 | 15.1 |
| 4 | 2 341 | 64 (45 - 74) | 38.5 | 2 165 | 16.0 |
| 5 | 2 649 | 64 (45 - 74) | 39.2 | 2 461 | 16.1 |
| 6 | 2 813 | 65 (45 - 74) | 39.3 | 2 608 | 15.5 |
| 7 | 2 990 | 65 (45 - 75) | 42.0 | 2 739 | 14.9 |
| 8 | 3 243 | 65 (49 - 75) | 41.5 | 3 014 | 14.7 |
| 9 | 3 319 | 65 (49 - 75) | 43.0 | 3 079 | 15.8 |
| 10 | 3 727 | 65 (50 - 75) | 43.1 | 3 494 | 14.3 |

Supplementary Table 3: Visual acuity (VA) outcome at one year of second treated eyes over deciles
